# Effect of different maternal metabolic characteristics on fetal growth in women with gestational diabetes mellitus

**Published:** 2013-04

**Authors:** Laleh Eslamian, Soheila Akbari, Vajihe Marsoosi, Ashraf Jamal

**Affiliations:** 1 *Department of Obstetrics and Gynecology, Tehran University of Medical Sciences, Tehran, Iran.*; 2 *Department of Obstetrics and Gynecology, Lorestan University of Medical Sciences, Khoramabad, Iran.*

**Keywords:** *Gestational diabetes mellitus*, *Macrosomia*, *Lipid*

## Abstract

**Background:** Fetal growth in diabetic pregnancies is a complex process and probably abnormalities in other metabolic pathways such as protein and lipid, as well as carbohydrate are responsible for delivering of macrosomic newborn.

**Objective:** The purpose of this study was to investigate the association between fetal growth and different maternal metabolic parameters in women with gestational diabetes mellitus (GDM) in comparison to control group.

**Materials and Methods: **This was a prospective cohort study conducted between March 2011 and May 2012, on 112 pregnant women with GDM and 159 healthy pregnant women. In order to determine of lipids or lipoproteins changes during pregnancy and to investigate any possible effects on fetal growth, lipid components, glucose and insulin levels were obtained in maternal serum three times in third trimester.

**Results:** Maternal serum glucose, total cholesterol (TC), low and high density lipoprotein (LDL-c, HDL-c) levels did not show any significant difference between two groups. While insulin, homeostasis model assessment-insulin resistance (HOMA-IR) and triglyceride (TG) values were detected to be significantly higher in the GDM cases especially after 32 weeks of gestation (p<0.001). After adjustment for confounding variables, maternal hypertriglyceridemia remained as a significant risk factor for delivering large for gestational age (LGA) newborns (p=0.04); and according to spearman test the increase of TG level was correlated with increase of insulin resistance and HOMA-IR (p<0.001, CI: 0.312).

**Conclusion:** Due to positive correlation of hypertriglyceridemia and hyperinsulinemia with newborn weight, it is possible to assume that elevated TGs levels in GDM cases is a reflection of variation in maternal insulin levels.

## Introduction

Diabetes mellitus is one of the most common medical complications during pregnancy (1-2). Gestational diabetes mellitus (GDM) is defined as any degree of glucose intolerance with onset or first recognition during pregnancy. Also it is associated with an increased risk for neonatal adverse outcome as well as maternal complications including preeclampsia and cesarean section (3-8). According to data, GDM occurs in 2-9% of pregnancies and this incidence in Iran has been reported 4.8% in 2005 (9, 10). 

It is reported that accelerated fetal growth and macrosomia are important factors for poor perinatal outcome in GDM (11). Macrosomia is a well-known indicator of maternal diabetes in fetus which is strongly associated with prematurity, respiratory distress syndrome, birth trauma, fetal death and adverse maternal outcome (3, 12-15). 

Probably it is due to some biochemical changes during the maternal hyperglycemia-fetal hyperinsulinemia pathways (3). It has been shown that maternal hyperglycemia which leads to fetal hyperglycemia, could affect pancreatic islet cells that consequently would cause fetal hyperinsulinemia. All the mentioned states results in increased liver glycogen contents, fat tissue and total body size (15-16). In recent decades, in spite of significant improvement in perinatal care, diagnosis, and treatment of GDM, macrosomia still remains as a serious complication which may involve up to 30% of diabetic pregnancies (12). 

It has been reported that the risk of newborns with large for gestational age (LGA) is increased five-fold in GDM and ten-fold in type-1 diabetes in spite of good glycemic control (17). Therefore it is suggested that gestational diabetes is associated with abnormalities in other metabolic pathways such as protein and lipid, as well as carbohydrate (18). The initial results obtained in previous studies led this project to evaluate plasma lipid levels in maternal serum (18-21). 

The purpose of this study was to investigate differences between maternal serum lipid levels in pregnant women with GDM and non-diabetic ones. Also the association among maternal serum lipid levels and fetal growth during late pregnancy and newborns birth weight were assessed in the current research.

## Materials and methods

According to routine of obstetric department of shariati hospital, all pregnant women receiving prenatal care at the obstetric clinic were screened using a 50 g oral glucose challenge test (OGCT) at 24-28 weeks of gestational age. Women with 1 hour glucose level of 140 mg/dl or more (positive screening OGCT) were given a 3- hour 100 g glucose tolerance test (OGTT) in order to diagnosis GDM. The test was performed in the morning after an overnight fast (8 hours fasting) while there has been at least 3 days of unrestricted diet (>150 gr carbohydrate/day) and exercises. 

According to Carpenter and Coustan criteria women who displayed two serum glucose concentrations (fasting ≥95 mg/dl, 1h>180 mg/dl, 2h>155 mg/dl, 3h>140 mg/dl) were diagnosed as GDM (22). Finally a total of 150 pregnant women were diagnosed with GDM between March 2011 and May 2012 in Shariati Hospital affiliated to Tehran University of Medical Sciences, Tehran, Iran. Exclusion criteria were a history of systemic underlying diseases (cardiovascular, renal, thyroid, liver, autoimmune and connective tissue disorder), substance abuser, overt diabetes mellitus (except previous history of GDM), multifetal gestations and major fetal malformation. 

After exclusion, 132 women with GDM were included. Of the including patients 112 women with complete maternal blood sample for glucose and lipid profile and ultrasound study reminded in the final analysis. As the control subjects, 200 healthy non diabetic pregnant women with singleton fetus were selected, among them only two women with chronic hypertension (n=2) were excluded. Finally 198 women were included and of them 159 women reminded in the final analysis, and also their newborns were selected and added to current research. 

All patients with GDM were prescribed medical nutrition treatment by a trained dietician. They were educated to self-monitoring of blood glucose levels by four times measurements a day or more according to need (fasting, two hours after meal). The objective was to maintain fasting glucose levels of 95 mg/dl or less and postprandial levels of 120 mg/dl or less according to American Diabetes Association (ADA) guidelines .Those who failed to achieve target glucose levels were treated with insulin. Insulin was started if two fasting blood glucose or one fasting glucose and postprandial value or two postprandial values were more than target glucose value. 

Women in the insulin group treated with NPH insulin as an intermediate-acting insulin with initial dose of 0.2 unit/kg if the fasting blood glucose value was high it was given before bedtime, if postprandial blood glucose was high, regular Insulin as a short-acting insulin was received before meals based on postprandial glucose level (1 unit for every 10 mg/dl over target value) and if both fasting and postprandial glucose level were high it was started a total dose of 0.7 unit/kg. The insulin was divided in two third as NPH insulin (given in two third 30 minute before breakfast and one third before bedtime) and either one third as two or three preprandial regular insulin injection as necessary.

**Maternal characteristics**

Using a combination of interviews and questionnaires in timing of glycemic screening (24-28 weeks), information on maternal characteristics such as age, gravidity, gestational age, educational levels, pregestational BMI, weight gain during pregnancy, blood pressure, family history of diabetes, hypertension and dyslipidemia were collected. 

**Blood sampling and tests**

Blood samples were collected at 28-32, 32-36 and 36 weeks of gestational age until delivery time in order to determine fasting serum levels of glucose, two hours postprandial levels of glucose in GDM group, TGs, total cholesterol, HDL-c, and insulin. LDL-c was calculated by the Friedewald formula (23) [LDL=TC–HDL–(TG/5)] and if TG >400 mg/dl, it was measured directly in serum. Glycosylated hemoglobin (HbA1c) was measured as percent of total hemoglobin at entry to the study in GDM group. 

**Ultrasound**

Fetal growth was measured by ultrasound at the same time as the maternal blood sample collecting. In order to check for abdominal circumstance (AC) and the estimate fetal weight, hadlock method was used (24). 

**Newborns characteristics**

Newborns data including gestational age at delivery time, birth weight (gr) and length (cm), gender, Apgar score (at 1 and 5 min), early preterm labor (gestational age ≤34 weeks), late preterm labor (gestational age >34 weeks and <37 weeks), small for gestational age (SGA: birth weight <10^th^ percentile), appropriate for gestational age (AGA: birth weight >10th and <90^th^ percentile), large for gestational age (LGA : birth weight >90th percentile) based on gestational age and sex were collected. Cut off values for fetal macrosomia were considered birth weight ≥4000 gr (25).

**Maternal outcome**

Maternal outcome such as mode of delivery, cesarean section rate, polyhydramnios and preeclampsia were recorded. Then fasting insulin levels and estimated insulin resistance by calculating the homeostatic model assessment (HOMA) index: fasting insulin (µu/ml) × fasting glucose (mg/dl) / 405 were measured. This study was approved by the Ethics Committee of Endocrinology and Metabolism Research Center (EMRC) in accordance with Helsinki declaration and guideline of Iranian Ministry of Health and Medical Education (code:E-00144) in Tehran University of Medical Sciences. 

**Statistical analysis**

All statistical analyses were performed using SPSS 16.0 for Windows software. The data on continuous variable with normal distribution were presented as mean±SD. Qualitative variables were shown as numbers and percentages. Comparisons between proportions were done using chi-square, Fisher exact, and the Anova tests. In the model, parity, age, history of GDM in previous pregnancy, family history of diabetes mellitus, prepregnancy BMI and weight gain during pregnancy were used as confounding variables. Also p<0.05 was considered statistically significant.

## Results

After exclusion of women with multifetal gestations (n=4), thyroid disorder (n=3), hypertensive disorder (n=5), overt diabetes (n=5), an connective tissue disorder (n=1), 132women with GDM were included. Of these, 112 women with complete maternal blood sample for glucose and lipid profile and ultrasound study reminded in the final analysis (lost to follow-up=20). As the control subjects, 198 women were included and of these 159 women reminded in the final analysis (lost to follow-up=39). There was no significant difference between lost to follow-up rate in both groups (15.2% vs. 19.7%, p=0.291). 

Only 8 women with GDM were treated with insulin and of them only one macrosomic baby was born. In these women the mean of insulin dosage at start of treatment was 6.2±4.5 IU/day and at the end of pregnancy was 14.2±9.5 IU/day. The mean of FBS was <95 mg/dl in about 79% of women in insulin group. About 81% of this group reached to goal of treatment in postprandial glucose checking (<120 mg/dl).

Maternal demographic characteristics at screening time are displayed in the table I and biochemical characteristics at 28-32w, 32-36w and 36 weeks of gestation until delivery time is also showed in table II. The gradual increase in serum lipid level variations during third trimester of pregnancy (28 weeks of gestation until delivery time) was observed in both groups. No significant difference was found in serum total cholesterol, LDL and HDL cholesterol levels at 28-32 weeks of gestation between two groups. The only significant different was detected in serum TG level. 

**Figure 1:**
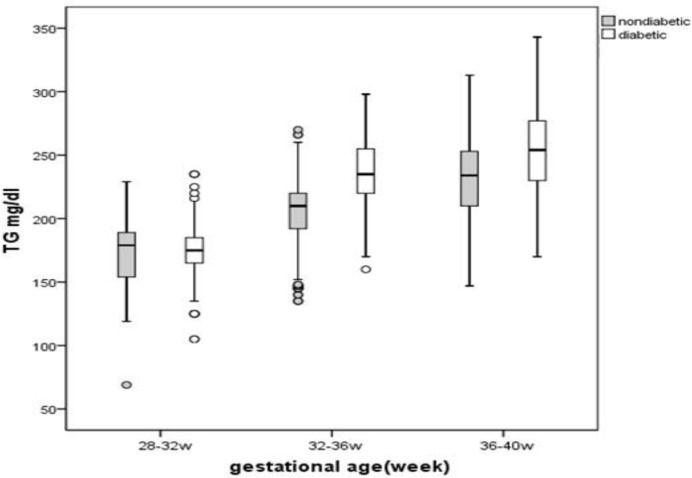
Comparison of serum TGs levels in women with GDM and control group in three different gestational ages. significant difference in 75th percentile of TG level at 32-36 weeks of gestation was observed

In GDM cases, TG levels in serum were increased in compression to control group at 32- 36 weeks of gestation (240.46±32.06 vs. 202.62±30.76, p<0.001). According to repeated measure test significant difference in serum TG level was started at 32- 36 weeks of gestation between two groups which has been shown in table II (p<0.001). Hypertriglyceridemia was defined as a condition in which serum TG level is more than the 75th percentile value. According to this definition significant difference in 75th percentile of TG level at 32-36 weeks of gestation was observed (Figure 1). 

According to Fisher exact test, there were no significant difference among cesarean rate (33.9% vs. 24.5%, p=0.06), polyhydramnios (7.1% vs. 3.8%, p=0.17) and preeclampsia (9.8% vs. 7.5%, p=0.327) in GDM group in comparison to control group. Neonatal outcome are shown in table III. Among all of GDM group, only one intra uterine fetal death was observed (at 34th week of gestation) which was caused by cardiomyopathy. Based on results for fetal growth monitoring using ultrasound (hadlock method) and also measurement of newborns birth weight, higher incidence rate of macrosomia and LGA in GDM group versus control group (17% vs. 7.5%, p=0.03 and 22.3% vs. 10.1%, p=0.009) was found.

**Figure 2:**
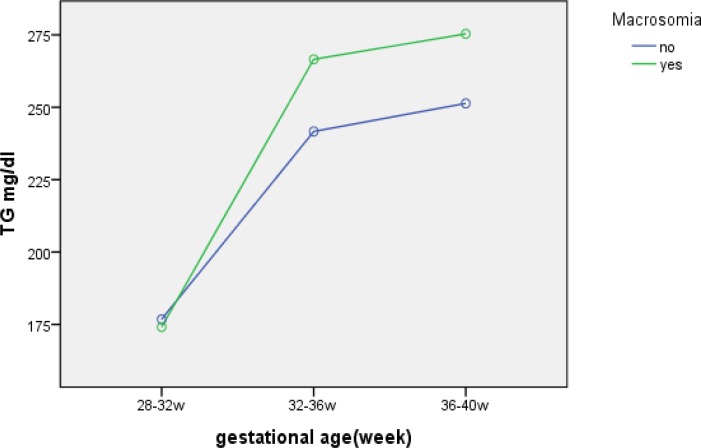
Association of gestational age and TGs levels in diabetic women with macrosomic and non macrosomic newborns a significant positive correlation between birth weight (macrosomia) and TG level in diabetic group after 32 weeks of gestational age was found

But there was no significant difference between prematurity rate in both groups (p=0.25). Based on to Bonferroni multiple comparison test, a significant positive correlation between birth weight (LGA and macrosomia) and TG level in diabetic group after 32 weeks of gestational age (p<0.001) was found (figure 2). But in non-diabetic group, TG level recorded lower in LGA group. For determination of independent prediction of birth weight in the study group adjustment analysis of covariance (ANCOVA) was performed. After adjustment for maternal pre-pregnancy BMI, weight gain during pregnancy, age, and parity, only TG level remind independently related to LGA (p=0.04). Although FBS levels were higher in GDM group but the mean of FBS was <95 mg/dl in 91% and the mean of two hours postprandial levels of glucose was <120 mg/dl in 85% of them. The mean of glycosylated hemoglobin concentration at screening time in GDM group was 4.9±0.32 mg/dl. 

For comparison of glucose effect on TG levels in two groups, fasting insulin levels was measured and estimation of insulin resistance was calculated by HOMA index in order to assay the association among TG and insulin resistance and macrosomia. As the results showed positive linear correlation between HOMA-IR (IR used instead of insulin resistance) and gestational time was observed in control and GDM groups with significantly increase in GDM group (1.73±0.526 vs. 2.75±1.05 at 28-32 w, 1.93±0.49 vs. 3.44±1.18 at 32-36w and 2.36±0.41 vs. 4.63±0.81 at 36-40w, p<0.001). 

**Figure 3:**
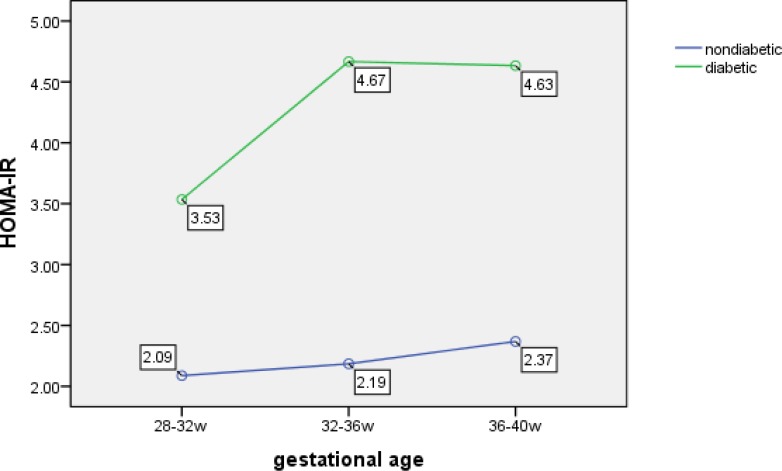
HOMA-IR in diabetic and control groups with macrosomic newborns. a significant increase of HOMA-IR in diabetic group versus control group was detected

The mean of maternal HOMA index was significantly higher in women with macrosomic newborns than in non macrosomic group (4.66±0.83 vs. 3.16±1.07, p=0.004) in diabetic pregnant women. After running 3 measurements of HOMA-IR test and comparing results in all the mothers with macrosomic newborns, a significant increase of HOMA-IR in diabetic group versus control group was detected (figure 3). According to spearman correlation coefficient test significant association between TG level and HOMA-IR was started at 32- 36w of gestation in diabetic group (p<0.001) (Table IV).

**Table I:** Baseline maternal characteristics at screening time

**Characteristics**	GDM**	**Control**	p-value**
Maternal age (year)	27.23 ± 4.19	25.48 ± 4.06	0.001
Parity	2.74(66.1)	2.94 (59.1)	0.325*
Previous birth weight (gr)	3301.32 ± 364	3210.31 ± 285	0.162*
Positive history of GDM [n(%)]	9(8%)	9(3.8%)	0.131*
Positive family history of diabetes [N(%)]	23 (20.5%)	9 (5.7%)	0.001
Prepregnancy weight (kg)	67.40 ± 10.00	59.55 ± 7.96	0.000
Gestational weight gain (kg)	13.82 ± 2.9	12.33 ± 3.4	0.20*
Baseline BMI (kg/m^2^)	26.59 ± 3.6	23.98 ± 2.6	0.000
Gestational age at entry time (wk)	27.02 ± 0.68	26.55 ± 1.08	0.000

GDM: gestational diabetes mellitus.           BMI: body mass index.

ns*: no significant (p<0.05).

**Table II:** Biochemical characteristics at 28-32, 32-36 and 36 weeks of gestation until delivery time

**Factors**		**28-32 w**	**32-36 w**	**36-40 w**	**p-value 1**	**p-value 2**	**p-value 3**
FBS							
	Non diabetic	69.27 ± 6.41	78.01 ± 6.24	80.08 ± 12.8	0.000	0.000	0.000
	Diabetic	84.50 ± 11.08	81.36 ± 10.5	84.76 ± 11.6			
TG							
	Non diabetic	170.32 ± 27.73	202.62 ± 30.76	229.33 ± 36.01	<0.001	<0.001	<0.001
	Diabetic	175.71 ± 24.23	240.46 ± 32.06	253.87 ± 39.61			
TC							
	Non diabetic	215.60 ± 22.45	238.58 ± 35.50	251.54 ± 34.36	0.09	0.657	0.918*
	Diabetic	218.90 ± 33.82	240.99 ± 29.44	254.24 ± 34.13			
HDL							
	Non diabetic	55.38 ± 3.10	59.66 ± 4.15	59.10 ± 4.03	0.07	0.1	0.1*
	Diabetic	55.37 ± 4.26	59.29 ± 4.61	59.35 ± 3.66			
LDL							
	Non diabetic	125.12±21.48	137.86±32.53	148.57±30.99	0.248	0.757	0.554*
	Diabetic	128.84±29.23	137.64±29.22	147.12±32.59			
LDL/HDL							
	Non diabetic	2.28 ± 0.38	2.33 ± 0.60	2.61 ± 0.59	0.084	0.377	0.312*
	Diabetic	2.31 ± 19	2.44 ± 0.53	2.62 ± 0.61			
Insulin							
	Non diabetic	6.64 ± 2.11	7.48 ± 2.17	8.01 ± 2.36	0.000	0.000	0.000
	Diabetic	12.8 ± 4.85	15.21 ± 4.91	16.18 ± 5.30			

FBS: Fasting Blood Sugar           TG: Triglyceride          TC: Total cholesterol           HDL: High Density Lipoprotein

LDL: Low Density Lipoprotein          Cholesterol in mg/dl,          Insulin in µu/ml,           ns*:no significant,

p_1:_: comparison of indices in diabetic and non diabetic patients according to repeated measure test.

p_2 _: comparison of indices in different gestational age according to analysis of covariance (ANCOVA).

p_3_: interaction effect of diabetes and gestational age on indices according to repeated measure test_._

**Table III:** Comparison of neonatal outcomes between study groups

		**Total (N=271)**	**GDM (N=112)**	**Control (N=159)**	**p-value**
Gestational age (wk)		38.1 ± 1.53	37.72 ± 1.7	39.36 ± 1.33	0.001
Weight (gr)		3291.29 ± 552	3336.07 ± 630	3259.75 ± 490	0.264*
Height (cm)		9.86 ± 051	49.54 ± 0.02	50.08 ± 0.01	0.13*
BMI (kg/m^2^)		13.17 ± 1.79	13.48 ± 2.02	12.95 ± 1.58	0.01
Apgar (1 min )		8.79 ± 0.75	8.67 ± 1.06	8.88 ± 0.396	0.02
Apgar(5 min )		9.86 ± 0.69	9.74 ± 1.02	9.94 ± 0.24	0.02
Male [N(%)]		143 (52.8%)	60 (53.6%)	83 (52.2%)	0.461*
Female [N(%)]		128 (47.2%)	52 (46.4%)	76 (47.8%)	0.53*
Macrosomia [N(%)]		31 (11.4%)	19 ( 17% )	12 (7.5 %)	0.03
LGA [N(%)]		41 (15.2%)	25 (23.3%)	16 (10.1%)	0.009
SGA [N(%)]		11 (4.1%)	6 (5.4%)	7 (4.3%)	0.171*
Prematurity [N(%)]					
	34 -37 W	19 ( 7%)	11 (9.8 %)	8 (5%)	0.255*
	<34 W	10 (2.7%)	5 (4.5 %)	5 (3.1%)	0.176*

LGA: large for gestational age.          SGA: small for gestational age. ns*: no significant(p<0.05)

**Table IV:** Correlation between TG levels and HOMA index in diabetic groups

** TG**		**28-32W**	**32-36W**	**36-40W**
**HOMA**				
**28-32W**				
	Correlation	0.036	-	-
	p-value	0.705	-	-
**32-36w**				
	Correlation	-	0.312	-
	p-value	-	0.001*	-
**36-40W**				
	Correlation	-	-	0.306
	p-value	-	-	0.002 *

HOMA: homeostatic model assessment.           TG: Triglyceride*spearman correlation coefficient test

## Discussion

In this prospective study, the association between different maternal plasma lipid and lipoprotein concentrations in GDM cases and normal pregnant women with pregnancy outcome, risk of over growth and macrosomia were investigated. The main findings can be summarized in the following points:

- A: Lipid level variations during 28 weeks of gestation to delivery time characterized by the gradual increase and peaking before the delivery in both groups (without significant variations in HDL cholesterol).
- B: There was no significant difference between total serum cholesterol, LDL cholesterol and HDL cholesterol in both groups based on gestational time.
- C: High levels of TGs during pregnancy after 32 weeks of gestation were observed in GDM group in comparison to control group.
- D: Only in diabetic group, maternal fasting hypertriglyceridemia after 32 weeks of gestation was a significant risk factor for delivering LGA or macrosomic newborns at term.
- E: The increase of TG levels during third trimester was correlated with increase of insulin resistance.

Even though hormonal changes in pregnancy have been associated with plasma lipid variation, is not yet understood the mechanism which pregnancy alters lipid metabolism. 

A decrease in lipoprotein lipase activity and an increase of hepatic lipase activity has been reported during pregnancy. Hepatic lipase is the responsible factor for the increase of triglycerides synthesis in the liver whereas in the adipose tissue, decreased of lipoprotein lipase lead to decrease of catabolism which in turn increases the serum triglyceride (26). 

As Wiznitzer *et al* reported, estrogen can induce hepatic production of very low density lipoprotein lipase (VLDL), which causes a gradual increase in lipids levels in second half of pregnancy in order to supply stable fuel supplementation to the fetus (27). Similar to the result of Wiznitzer *et al* we have found that lipids levels are increased in second half of pregnancy and peaking before delivery in both groups. 

There are some investigations about lipid profile in gestational diabetes. According to the published investigations hyperlipidemia such as hypertriglyceridemia is pronounced in GDM pregnancies (18, 28, 29). In another study it was concluded that pregnant women with GDM have higher serum triacylglycerol but lower LDL cholesterol concentrations in comparison to normal pregnancy (20). On the other hand, a research has shown that total cholesterol, HDL cholesterol, and APO lipoprotein concentrations are not significantly different between GDM and control subjects (30). Montelongo *et al* reported that there were no difference in triglyceride, VLDL, LDL and HDL cholesterol concentrations between women with GDM and controls throughout the pregnancy (31, 32).

In this study, analysis of patients with GDM showed an increase of TG during the third trimester of pregnancy especially after 32 weeks of gestation which was correlated with increased levels of insulin. This finding was similar to Wiznitzer *et al* study (27). It has been reported that an anti- lipolytic effect of insulin could decrease maternal FFA and TG levels and also leads to reduction of fetal fat mass (33). However increase in estrogen levels and insulin resistance seems to be responsible for the pregnancy hypertriglyceridemia (30).

 Macrosomia is a frequent complication of pregnancy in diabetes. It was reported that fetus of an insulin dependent diabetic mother has a different growth pattern in comparison to a fetus of healthy mother. A biphasic fetal growth was seen with growth retardation in the first and second trimester and growth acceleration in the third trimester as a result of fetal hyperinsulinemia (34). The incident of LGA newborns in current studied group was approximately 19% which is similar to findings of some other studies (11, 32, 35).

According to the data, high TGs levels are associated with higher birth weight, as might be expected in insulin resistance state (27). Our findings seem to be in accordance with Kitijoma *et al* and Knopp *et al* results which shows that high maternal TG levels in third trimester could be a predicting factor for the macrosomia (29, 36). In this study the association between hypertriglyceridemia and macrosomia at 32 weeks of gestation was observed that correlated with increase of insulin levels.

Published data have been limited to a one-time point measurement in conjunction with OGTT, whereas this study was similar to the study of Schaefer Graf *et al* considers lipid and glucose data at different levels during the third trimester, in addition a control group was used in this study for better and effective comparison (33). 

In the present study, fasting insulin levels and estimated insulin resistance was calculated by HOMA index. As the results showed positive linear correlation between HOMA-IR and gestational time was observed in control and GDM groups with significant increase in GDM group which was similar to Negrato *et al* study (37). The mean of maternal HOMA index was significantly higher in the macrosome group compared to the non macrosome ones which was similar to Son *et al* study (18).

Also a positive correlation between serum insulin levels and HOMA-IR in GDM subjects and macrosomia was observed, as well as between maternal serum TG levels and macrosomia. Thus these findings are able to support this hypothesis which explains insulin resistance could be an important factor in elevation of TG levels and fetal weight. This may be an evidence for the study of Wiznitzer et al. that reported the association between GDM and high TGs levels supports the insulin resistance theory (27).

## Conclusion

Fetal growth in diabetic pregnancies is a complex process and other maternal metabolic factors are responsible for delivering of LGA newborns such as glucose and insulin levels. In this study, in diabetic mothers with LGA infants, an increased concentration in TG was observed which is suggested that TGs might be involved in regulation of birth weight. Due to positive correlation of hyperinsulinemia and hypertriglyceridemia in third trimester with newborn weight at term, it is possible to assume that elevated TGs levels in GDM cases is a reflection of variation in maternal insulin levels. These observations indicated that measurement of plasma TGs may be valuable in prognosticating fetal outcome and in identifying LGA and macrosomia in gestational diabetes. Therefor In addition to measurement of serum glucose levels, other measurements seem to be necessary to reduce the risk of macrosomia in this group of patients.

This study had several limitations. Due to the small sample size, significant association between pregnancy complication such as preeclampsia and polyhydramnios in both groups were not observed. The major difficultly encountered during this research was the lack of official reference values for lipid adapted in pregnancy. Finally, it is difficult to compare and contrast lipids value from different studies, because maternal characteristics, socioeconomic state, foods and nutrition, lifestyle and mode of delivery are heterogeneous.
